# The impact of obesity on walking and cycling performance and response to pulmonary rehabilitation in COPD

**DOI:** 10.1186/1471-2466-10-55

**Published:** 2010-11-06

**Authors:** Francesco Sava, Louis Laviolette, Sarah Bernard, Marie-Josée Breton, Jean Bourbeau, François Maltais

**Affiliations:** 1 Centre de recherche, Institut Universitaire de Cardiologie et de Pneumologie de Québec, Université Laval, Québec, Canada; 2 Respiratory Epidemiology and Clinical Research Unit, Montreal Chest Institute of the Royal Victoria Hospital, McGill University Health Centre, McGill University, Montreal, Canada

## Abstract

**Background:**

We examined the influence of overweight and obesity on pulmonary function, exercise tolerance, quality of life and response to pulmonary rehabilitation in COPD.

**Methods:**

261 patients with COPD were divided into three groups: normal body mass index (BMI), overweight and obese. Baseline and post rehabilitation pulmonary function, 6-min walking test (6MWT), endurance time during a constant workrate exercise test (CET) and St. George's Respiratory Questionnaire (SGRQ) scores were compared between all three classes of BMI.

**Results:**

At baseline, obese and overweight patients had less severe airflow obstruction compared to normal BMI patients. There was no baseline difference in CET performance or SGRQ scores across BMI classes and 6MWT was reduced in the presence of obesity (p < 0.01). Compared to baseline, post-rehabilitation 6MWT, CET performance and SGRQ scores improved significantly in each group (p < 0.01), but 6MWT was still significantly lower in the presence of obesity.

**Conclusions:**

Walking, but not cycling performance was worse in obese patients. This difference was maintained post rehabilitation despite significant improvements. Weight excess may counterbalance the effect of a better preserved respiratory function in the performance of daily activities such as walking. However, obesity and overweight did not influence the magnitude of improvement after pulmonary rehabilitation.

## Background

Chronic obstructive pulmonary disease (COPD) is associated with dyspnea and exercise intolerance, two major impediments to quality of life. Although low body weight[1] and muscle wasting[2] have traditionally been the focus of nutritional management in COPD, recent data indicate that obesity is becoming frequent in this disease[3]. On one hand, a high body mass index (BMI) appears to convey a survival advantage to patients with COPD[1,4]. On the other hand, obesity by itself may compromise lung function[5], decrease exercise tolerance particularly during weight bearing activities[6,7], and quality of life[8], leading to greater disability[9,10].

The effects of obesity in combination with COPD on exercise tolerance and dyspnea have received little attention. In one study, obese patients with COPD had a greater peak exercise capacity and reduced dyspnea perception at a standardized ventilation during incremental cycling exercise compared to their lean counterparts[11]. These counterintuitive beneficial effects of obesity were felt to be related to reduced operating lung volumes during exercise in the obese individuals. Other studies showed that the 6-min walking distance [3], but not constant exercise cycling test time[12], was reduced in obese patients with COPD compared to non-obese patients highlighting the importance of taking into account the exercise testing modality before concluding about the impact of obesity on exercise capacity in COPD. Whether overweight may also influence exercise capacity in COPD has not yet been addressed.

Pulmonary rehabilitation addresses the systemic consequences of COPD, beyond the impairment in lung function. As summarized in a recent meta-analysis[13] pulmonary rehabilitation improves dyspnea, exercise tolerance and quality of life. Because of the growing prevalence of weight excess in COPD[14], it is important to learn about the impact of overweight and obesity on pulmonary rehabilitation. A retrospective study[3] showed that obesity did not adversely affect rehabilitation outcomes, although data obtained prospectively would be useful to confirm these findings.

Based on the existing data suggesting that overweight and obesity may interact with COPD, our hypothesis was that increasing BMI in COPD would reduce exercise tolerance, increase exertional dyspnea and reduce functional status during walking but not cycling and compromise the response to pulmonary rehabilitation in patients with COPD. This study was thus undertaken to investigate the effects of overweight and obesity combined with COPD on 1) resting pulmonary function; 2) 6-min walking distance and endurance time during a constant workrate cycling exercise test (CET time) 3) health-related quality of life and 4) improvement of these parameters following pulmonary rehabilitation. To address these issues, we took advantage of a prospective cohort of patients with COPD entering pulmonary rehabilitation in Canada.

## Methods

### Study participants

Patients with COPD about to take part in pulmonary rehabilitation were recruited in 10 study centers across Canada. Inclusion criteria were: stable COPD, post-bronchodilator forced expiratory volume in one second (FEV_1_) <70% predicted and FEV_1_/forced vital capacity (FVC) <70%. Exclusion criteria were: participation to pulmonary rehabilitation in the preceding 12 months, living in a long term care facility and a diagnosis of asthma, congestive heart failure or dementia. All patients gave informed consent to participate in the study. Ethics committee from all 10 study sites approved this research project.

### Study design

The data for this study was collected as a part of a prospective observational study of pulmonary rehabilitation in Canada. The main objective of this cohort was to compare home versus hospital-based pulmonary rehabilitation[15]. The secondary objectives were to identify possible predictors of the response to pulmonary rehabilitation, including obesity, and to evaluate the responsiveness of different evaluative tools to assess the effects of pulmonary rehabilitation[16]. The participating centres agreed on a pre-established research protocol describing the evaluation process that was standardized and performed by qualified study personels. Study monitoring was ensured by one of the author (SB). The length of the pulmonary rehabilitation programs (6 to 12 weeks) could not be standardized because of different rehabilitation capacity between centres. Patients' assessment included a medical history, pulmonary function tests, and CET, 6MWT and health status measured by the St. George's Respiratory Questionnaire (SGRQ). Dyspnea at rest was evaluated with the MRC dyspnea score[17]. Data was collected at baseline and immediately after the pulmonary rehabilitation program. Patients were classified according to BMI classification of the World Health Organization[18] into normal BMI (BMI 18.5 - 24.99 kg·m^-2^), overweight (25 - 29.99 kg·m^-2^) and obese (>30 kg·m^-2^).

### Pulmonary function

Spirometry and lung volumes were measured according to recommended procedures[19]. Results were compared with predicted normal values from the European Respiratory Society[20]. Disease severity was categorized according to the Global Initiative for Chronic Obstructive Lung Disease (GOLD) classification system[21].

### Constant workrate cycling exercise test (CET)

CET was performed on a cycle ergometer with a workload set at 80% of peak work capacity achieved during incremental cycle ergometry. Patients were asked to cycle for as long as possible[22]. The minimum clinically important difference (MCID) in exercise time was set at 100 s[16].

### Six-minute walking test (6MWT)

The 6MWT was administered in an enclosed corridor in accordance to the procedures recommended by the American Thoracic Society (ATS)[23]. The MCID in walking distance was set at 54 m[24]. We also calculated the body weight-walking distance product in m·kg (walk-work) at baseline[25].

### Health status

Health status was evaluated using French or English versions of the SGRQ[26]. This disease-specific questionnaire has been extensively validated in patients with all grades of respiratory disease including advanced COPD[27]. A score change of 4 points was considered clinically significant[28].

### Symptoms assessment

Ratings of perceived exertion were reported by patients at the end of exercise tests (CET and 6MWT) on a 10-point Borg scale, for dyspnea and leg fatigue. The MCID for Borg scores was set at 1 unit[29].

### Pulmonary rehabilitation

Rehabilitation program consisted of 6 to 12 weeks of tri-weekly 90-minute exercise sessions that integrated stationary bicycle endurance training, resistance exercises, and patient education, which has been described extensively elsewhere[15,30]. The exercise training program was directly supervised (n = 190) or was delivered at home (n = 71). Since these two interventions gave similar results on dyspnea, quality of life and exercise tolerance[15], data from these two training strategies were combined in the present study.

### Statistical analysis

Results are reported as mean ± SD. A p value < 0.05 was considered as statistically significant. One-way ANOVA was used to compare baseline characteristics, except gender for which we used the Pearson's chi-square. Post rehabilitation data was compared to baseline using repeated measures two-way ANCOVAs (group, intervention) using baseline spirometric data and lung volumes as covariates (FEV_1_% predicted, FEV1/FVC ratio, functional residual capacity (FRC)% predicted, residual volume (RV)% predicted, total lung capacity (TLC), inspiratory capacity (IC)/TLC ratio). The normality assumption was verified using the Shapiro-Wilk's statistic while the homogeneity of variances was verified graphically with the residuals plot. Univariate and multivariate regression analyses were carried out to identify possible correlates of the response to pulmonary rehabilitation using age, sex, BMI, length of rehabilitation program and all the pulmonary function tests reported in table 1 as independent variables. All the analyses were done using SAS software, release 9.2 (SAS Institute inc., NC).

**Table 1:** Baseline characteristics of patients

	Normal BMI	Overweight	Obese	ANOVA
	n = 88	n = 95	n = 78	p-value
Age, years	66.0 ± 9.4	65.7 ± 8.2	65.4 ± 8.0	NS
Sex, % of men	56	61	59	NS
Body mass index, kg/m^2^	22.3 ± 1.8*	27.5 ± 1.5*	35.1 ± 3.5	<0.001
Height (cm)	164 ± 9.1	165 ± 9.2	163 ± 9.8	NS
Weight (kg)	61 ± 8.6*	75 ± 9.8*	91 ± 14.6	<0.001
BODE score	3.9 ± 2.0*	3.1 ± 2.0	3.2 ± 2.4	0.002
**Pulmonary function**				
FEV_1_, L	1.02 ± 0.39*	1.18 ± 0.42	1.14 ± 0.38	0.002
% predicted	42 ± 15*	49 ± 15	49 ± 17	0.002
FVC, L	2.7 ± 0.8	2.6 ± 0.8	2.5 ± 0.9	NS
% predicted	86 ± 22	83 ± 21	82 ± 23	NS
FEV_1_/FVC, %	40 ± 10*	45 ± 12*	49 ± 12	0.001
FRC, L	5.0 ± 1.6†	4.4 ± 1.5	4.1 ± 1.7	<0.001
% predicted	159 ± 42†	142 ± 41	135 ± 45	<0.001
RV, L	4.2 ± 1.4*	3.6 ± 1.4	3.5 ± 1.5	0.002
% predicted	178 ± 60*	157 ± 58	151 ± 64	0.006
TLC, L	6.8 ± 1.7*	6.3 ± 1.7	6.1 ± 1.8	0.01
% predicted	121 ± 22*	112 ± 21	110 ± 23	0.001
IC, L	1.8 ± 0.6	2.0 ± 0.6	2.0 ± 0.6	NS
% predicted	73 ± 21	77 ± 20	81 ± 19	NS
IC/TLC, %	28 ± 8*	32 ± 9	33 ± 8	<0.001
**Incremental exercise test**				
Peak $\dot{V}O_{2}$, L·min^-1^	0.85 ± 0.29*	1.02 ± 0.32*	1.12 ± 0.40	0.02
Peak $\dot{V}O_{2}$, % predicted	61 ± 36*	75 ± 56*	87 ± 69	0.01
Peak work capacity, Watt	59 ± 22	69 ± 29	65 ± 29	NS
Peak work capacity, % predicted	57 ± 28	64 ± 41	64 ± 37	NS
**Constant exercise test**				
CET time, s	367 ± 230	324 ± 201	338 ± 203	NS
**6-minute walking test**				
6MWT, m	407 ± 75	391 ± 78	342 ± 79*	0.001
Work-walk, m·kg	23296 ± 5902*	29590 ± 8045*	32779 ± 11078*	<0.001
**Quality of life**				
SGRQ total score	45 ± 17	45 ± 16	45 ± 18	NS
SGRQ symptoms score	53 ± 23	51 ± 22	50 ± 24	NS
SGRQ activity score	65 ± 20	65 ± 20	65 ± 22	NS
SGRQ impact score	32 ± 18	32 ± 17	32 ± 19	NS
**Pulmonary rehabilitation**				
Duration of rehabilitation (weeks)	8.5 ± 1.7	8.5 ± 1.7	8.4 ± 1.7	NS
Home based rehabilitation, n (%)	23 (26)	27 (28)	21 (27)	NS

Values are mean ± SD. Definitions of abbreviations: FEV1: forced expiratory volume in 1 second; FVC: forced vital capacity; FRC: functional residual capacity; RV: residual volume; TLC: total lung capacity; IC: inspiratory capacity; $\dot{V}O_{2}$: oxygen consumption; CET: constant exercise test; 6MWT: 6-minutes walking test distance; SGRQ: St-George's respiratory questionnaire; NS: not significantly different. * = significantly different than the other two groups, p < 0.05. † = significantly different than obese group, p < 0.05.

## Results

### Baseline characteristics

**Figure 1:**
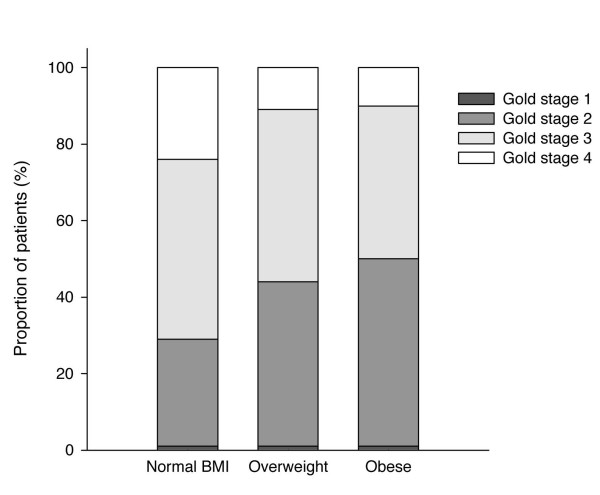
**GOLD stage distribution across body mass index (BMI)**.

Three hundred patients were initially enrolled in the present study. A 13% drop-out rate was observed during pulmonary rehabilitation. The drop-outs were evenly distributed among the three groups. Reasons for dropping-out were: patient withdrawal (11%), lost to follow-up (1.5%) and death (0.5%). We report here data for the 261 patients who have completed pulmonary rehabilitation and whose baseline characteristics are presented in table 1. Patients had a mean FEV_1 _of 46 ± 15% of predicted value. GOLD stage distribution was as follows: stage 1, 1% of the total population; stage 2, 40%; stage 3, 44%; and stage 4, 15% (figure 1). Mean age was 65 ± 8 years and 57% of patients were males. There were no patients with BMI under 18.5 kg/m^2 ^and only 5 patients with BMI >40 kg/m^2^.

Sixty percent of the study population was either obese or overweight, a proportion reflective of the Canadian population aged 40 years or older[31]. FEV_1 _(L and %), FEV_1_/FVC ratio and inspiratory capacity (IC) to TLC ratio (IC/TLC) were significantly lower in the normal BMI group than the other two groups (p < 0.05). Residual volume (RV, L and %), total lung capacity (TLC, L and %), and BODE scores[4] were significantly higher in the normal BMI group than in the other two groups (p < 0.05). Functional residual capacity (FRC, L and %) was significantly higher in the normal BMI group than in the obese group. There was a larger proportion of GOLD stage II patients in the overweight and obese groups (figure 1).

**Figure 2:**
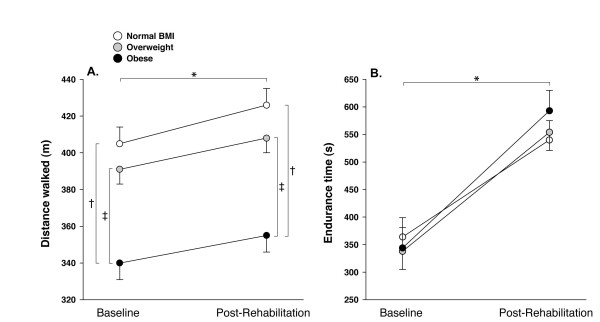
**6-minute walking distance and endurance time to constant workrate exercise according to the 3 body mass index (BMI) categories, at baseline and after pulmonary rehabilitation**. Values are mean (SD). * = p < 0.01 baseline versus after rehabilitation within each BMI group, † = p < 0.01 obese versus normal BMI, ‡ = p < 0.01 obese versus overweight.

At baseline, 6MWT distance in the obese group was 65 m shorter compared to normal BMI (p < 0.01) and 49 m shorter compared to overweight (p < 0.01) (table 1 and figure 2). Work-walk at baseline was significantly higher in the obese group (32779 ± 11078 m·kg, p < 0.01) compared to the other two groups and was higher in the overweight group (29590 ± 8045 m·kg) compared to normal BMI (23296 ± 5902 m·kg, p < 0.01). At baseline, CET time was similar across all BMI categories (p = 0.8) (table 1 and figure 2).

**Figure 3:**
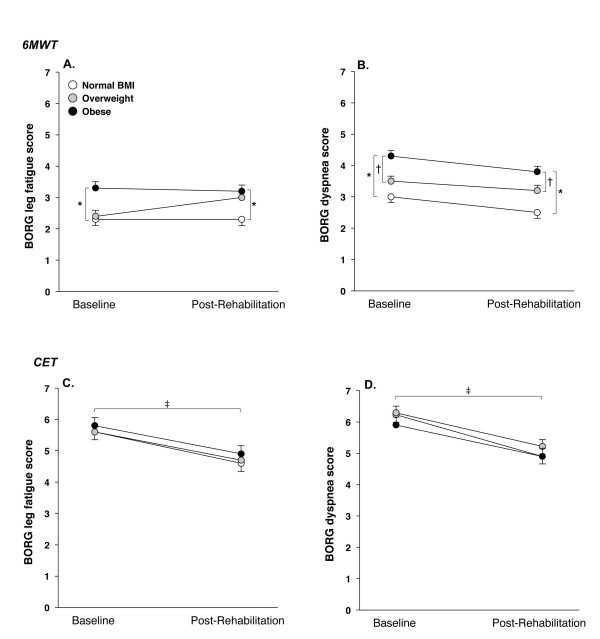
**Dyspnea and leg fatigue Borg scores at the end of the 6-minute walking test (6MWT) and of constant workrate cycling exercise (CET) according to the 3 body mass index (BMI) categories, at baseline and after pulmonary rehabilitation**. Values are mean (SD).). * = p < 0.01 obese versus normal BMI, † = p < 0.01, obese versus overweight, ‡ = p < 0.01 baseline versus after rehabilitation within each BMI group.

Borg dyspnea and leg fatigue scores after 6MWT were higher in the obese group at baseline (p < 0.05) (Figure 3a and 3b). During CET, Borg dyspnea and leg fatigue scores were similar between groups. Baseline SGRQ total scores were not significantly different between groups (table 1).

### Effects of pulmonary rehabilitation according to BMI

**Figure 4:**
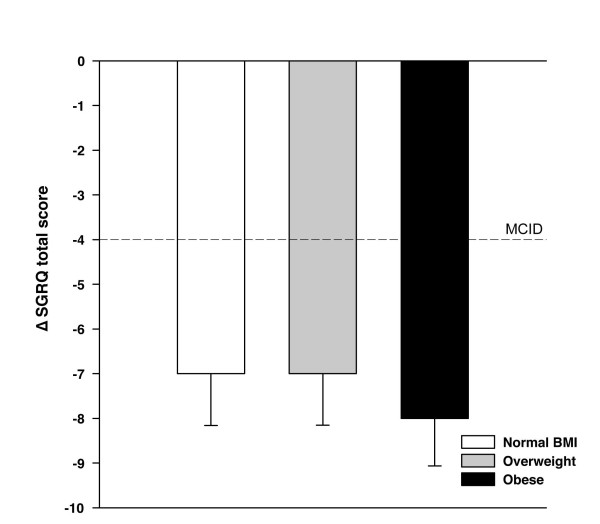
**Changes in St. George's Respiratory Questionnaire (SGRQ) total score with pulmonary rehabilitation according to BMI categories**. The horizontal dashed line represents the minimally important clinical difference (MCID) for this variable (∆ of 4 units). Values are mean (SD).

The duration and modality (home versus hospital-based) of pulmonary rehabilitation programmes were similar across the three BMI categories (table 1). Albeit small, the pre versus post rehabilitation difference in the 6-min walking distance were statistically significant (p < 0.01) and of similar magnitude within each group (mean 15-21 m, p = 0.92) (figure 2). Improvement in CET time following rehabilitation was also similar in the 3 groups and reached the clinical and statistical thresholds within each group (mean 175-216 seconds, p < 0.01) (figure 2). There was no significant reduction in 6MWT Borg scores with rehabilitation within the 3 BMI categories (figure 3a and 3b). This is in contrast to CET Borg scores and SGRQ scores which were significantly reduced (1.0-1.3 points, p < 0.01 and 7-8 points, p < 0.01 respectively) after rehabilitation in all three BMI groups (figure 3, panel c and d, and figure 4). In univariate and multivariate regression analyses, the changes in 6MWT distance, CET time and SGRQ scores as dependant variables were not statistically associated with BMI nor with any of the potential correlates of the response to rehabilitation that are outlined in the statistical analysis section.

MCID for the 6MWT, CET and SGRQ was reached by 19%, 46%, and 60% respectively in the entire study population. Table 2 shows the proportional MCID attainment according to BMI category. This proportion for the 6MWT was smaller for obese than normal BMI (15% vs 24%, p < 0.01) but was similar across groups for CET, SGRQ and Borg scores.

**Table 2:** Proportion of subjects reaching the MCID in each group for different outcomes

	Normal BMI	Overweight	Obese
6MWT, %	24*	18	15
CET, %	42	46	57
SGRQ, %	59	64	56
Dyspnea score at the end of 6MWT	46	37	46
Leg Fatigue score at the end of 6MWT	41	31	40
Dyspnea score at the end of CET	60	58	58
Leg fatigue score at the end of CET	56	51	60

Values are % of subjects reaching the MCID defined earlier on each group for three different outcomes. Definitions of abbreviations: 6MWT: 6-minutes walking test; CET: constant exercise test; SGRQ: St. George's respiratory questionnaire. * = p < 0.05 for normal BMI vs obese

The changes in BMI after rehabilitation were small and not statistically significant averaging -0.03 ± 0.98 kg/m^2 ^(range: -6.26 to 3.94 kg/m^2^). There was a significant reduction in BODE scores in the 3 groups with rehabilitation with a post-rehabilitation BODE scores of 3.1 ± 1.8, 2.5 ± 1.6, 2.6 ± 1.6, for normal BMI, overweight and obese, respectively, p < 0.01 versus pre-rehabilitation.

## Discussion

This study reports on the impact of obesity and overweight in a large prospective cohort of patients with COPD participating in pulmonary rehabilitation. The results can be summarized as follow: i) obese and overweight patients had higher FEV_1_, lower static lung volumes and higher peak incremental exercise capacity at baseline, ii) despite this, their CET time was not longer than that of patients with normal BMI, iii) obese patients had a reduced walking capacity compared to overweight and normal BMI patients, iv) BMI did not seem to affect SGRQ scores in the present population, finally v) overweight and obesity did not reduce the magnitude of improvement in exercise capacity and quality of life after pulmonary rehabilitation and BMI had no effect on outcomes on univariate or multivariate regression analyses taking account differences in baseline pulmonary function.

It is interesting to observe that, in this cohort, the proportion of overweight and obese patients was greater than normal BMI patients, a likely reflection of the obesity epidemic that afflicts industrialized countries [32,33]. These results underscore that the study of the impact of obesity and overweight in patients with chronic respiratory disorders will be a topic of interest in the coming years.

Obese and overweight patients had higher FEV_1 _and FEV_1_/FVC ratio than their lean counterparts, an observation that was previously reported [3,4,34,35]. One possible explanation is that patients with weight excess tend to be more dyspneic for a given FEV_1_[36] as illustrated by the higher Borg dyspnea and leg fatigue scores found in the obese patients during 6MWT. Therefore, obese patients with COPD might attract medical attention at an earlier stage of their disease. It is intriguing to consider that obesity may influence the natural history of COPD; in a subanalysis of the TORCH trial[37], BMI >25 kg/m^2 ^was associated with a slower decline in FEV_1_. Another possibility for the differences in baseline lung function relates to the influence of obesity on ventilatory function. Decreased chest wall and lung compliance in obesity[11] would tend to increase expiratory flows and decrease resting lung volumes.

At baseline, resting hyperinflation was reduced and the IC/TLC ratio increased in the obese population. This finding is consistent with those of Ora et al.[11]. One novel finding of our study is that overweight was also associated with reduced lung volumes in comparison with patients with normal BMI.

We found that obesity had a significant impact on walking capacity but not on the endurance time during cycling exercise. This is likely the result of the increase in energy expenditure associated with weight bearing exercise[38] as shown by higher body weight-walking distance product[25]. From a functional point of view, walking better represents daily activities than cycling. Taken together, these data suggest that obese COPD patients might have more important functional impairments. It would be interesting to study the impact of weight reduction strategies on walking capacity in obese patients with COPD.

As indicated by similar SGRQ total scores, there was no difference in health status between groups, both at baseline and post-rehabilitation, even though patients in obese group had a more limited walking capacity. This could be related to the fact that obese patients might compensate by adapting their environment and diminishing the amount of activity they perform.

In a retrospective analysis, Ramachandran and colleagues[3] reported that the improvement in 6-min walking distance and quality of life improved to a similar extent after rehabiliation in obese patients with COPD when compared with patients with a BMI < 30 mg/kg^2^. One limitation of that study is that it did not include overweight patients. This appears to be relevant given that overweight is even more common than obesity. Our prospective study therefore adds to this information in showing that dyspnea, quality of life and exercise tolerance improve as much in the obese and overweight COPD patients as their normal BMI counterparts. BODE scores improved significantly in our population within each group to an extent that is consistent with the literature[39].

We did not observe significant reductions in BMI after pulmonary rehabilitation. Exercise in itself is usually not sufficient to adequately manage obesity[40] and it should be done in conjunction with nutritional counseling which was not offered here. In the future, it will be important to learn how to intervene efficiently with COPD patients in their goal of loosing excess fat.

This study provides some novel information. First, it is, to our knowledge, the only study looking prospectively at the effect of BMI on pulmonary rehabilitation outcomes. We also report on improvements in terms of MCID for 3 different outcomes, the 6 MWT, CET and SGRQ. Although it is generally suspected that walking capacity is compromised in obese COPD patients, this study is the first to systematically investigate the impact of obesity on specific exercise modalities. Finally, the number of patients enrolled in our trial also provides sufficient statistical power to make valid conclusions.

The impact of comorbid conditions on rehabilitation outcomes is currently being investigated [41,42]. In general, it is felt that comorbidities do not prevent pulmonary rehabilitation from being effective although some conditions such as metabolic diseases and osteoporosis may reduce the chances of success [41,42]. The present study extends these results by showing that obesity reduces the likelihood of a patient achieving the MCID of improvement in distance walked during the 6MWT after rehabilitation. In contrast, the proportion of patients reaching the MCID for cycle exercise and SGRQ was not influenced by BMI. The proportion of our patients reaching the MCID for the SGRQ is similar to what has been reported [41]. Although we did not record the amount of aerobic and resistance training that was performed during rehabilitation, BMI was not a factor in the choice of the training strategies and modalities (home versus hospital-based) used in the three groups. It is thus unlikely that intrinsic differences in the design of the training programs were the main factors in explaining the lower proportion of obese patients reaching the MCID for the 6MWT.

Although the improvement in 6MWT following pulmonary rehabilitation was less than typically reported[41,43], we felt that the CET data was reassuring about the exercise enhancing effects of our rehabilitation programs. Measuring the cycling endurance time is a better test of the functional effect of pulmonary rehabilitation than the 6MWT[16]. The modest gain in the distance covered during the 6MWT probably reflects our program's emphasis on the bicycling component of the training intervention since the training modality is known to impact on specific outcomes. For example, patients training solely on stationary bicycle have less improvement in walking capacity compared to patients performing walking exercises[44].

Our study has potential limitations. First there were only 3% of morbidly obese patients (BMI >40 kg/m^2^), it would be important to see how such patients fare in the context of pulmonary rehabilitation. Secondly, it is well recognized that reduced fat free mass is associated with muscle weakness[45], decreased exercise tolerance[46] and poorer survival[47,48] in COPD. Reduced fat-free mass may occur despite normal BMI[49]. Thirdly, the CET and 6MWT dyspnea and leg fatigue Borg scores were collected only at the end of exercise. Dyspnea and leg fatigue scores obtained at isotime would have been useful to assess the effects of pulmonary rehabilitation on these variables in a more complete fashion. Finally, the impairment in baseline FEV_1 _was greater in patients normal BMI and the question may be raised as to whether this difference in disease stage between groups could explain our results. We do not believe that this is the case for the following reasons: *i) *the main analysis consisted in an analysis of covariance that took into account any differences in FEV_1 _and lung volumes at baseline; *ii) *since obese patients had milder airflow obstruction and resting hyperinflation, their performance during the 6MWT should have been better, not worse; and *iii) *the magnitude of improvement following pulmonary rehabilitation is independent from baseline lung function[50].

## Conclusion

Obesity and overweight are frequently associated with COPD. As the prevalence of excess weight is increasing, this association will be more and more frequent in clinical practice. Obese patients with COPD, despite having less severe airflow obstruction, resting hyperinflation and better peak VO_2 _than normal BMI patients, had more severe walking impairment. Pulmonary rehabilitation was still beneficial in improving several clinical outcomes despite the presence of excess weight. It would be of great interest to study the impact of weight reduction strategies in conjunction to exercise training in this specific patient population.

## Competing interests

L. Laviolette is recipient of a research training award from the Centre de Recherche de l'Institut Universitaire de Cardiologie et de Pneumologie de Québec. J. Bourbeau is the recipient of a John R. & Clara Fraser Memorial Award from the faculty of Medicine, McGill University. This work was supported by the Respiratory Health Network of the FRSQ and by a grant from GlaxoSmithKline, Canada. F. Maltais holds a GSK/CIHR Research Chair on COPD at Université Laval.

## Authors' contributions

FS and LL carried out the analysis of the database and drafted the manuscript. SB and MJB recruited the patients, performed the exercise tests and the questionnaires and help revising the draft. JB and FM conceived of the study, and participated in its design and coordination and helped to draft the manuscript. All authors read and approved the final manuscript.

## Pre-publication history

The pre-publication history for this paper can be accessed here:

http://www.biomedcentral.com/1471-2466/10/55/prepub
